# Mean platelet volume as a potential prognostic marker in patients with acute mesenteric ischemia–retrospective study

**DOI:** 10.1186/1749-7922-8-49

**Published:** 2013-11-25

**Authors:** Fatih Altintoprak, Yusuf Arslan, Omer Yalkin, Yener Uzunoglu, Orhan Veli Ozkan

**Affiliations:** 1 Department of General Surgery, Faculty of Medicine, Sakarya University, Sakarya, Turkey; 2 Department of General Surgery, Research and Educational Hospital, Sakarya University, Sakarya, Turkey

**Keywords:** Acute mesenteric ischemia, Mean platelet volume, Prognosis

## Abstract

**Introduction:**

We investigated prognostic parameters of patients who underwent surgical intervention for acute mesenteric ischemia by evaluating demographic characteristics and laboratory data on admission.

**Methods:**

The hospital records of 30 patients who underwent surgical interventions due to acute mesenteric ischemia between January 2008 and December 2012, were reviewed retrospectively. The records were investigated with regard to demographic data, the presence of co-morbid diseases, presenting complaints, time elapsed between symptom onset and hospital admission, laboratory findings at admission, findings at surgical exploration, surgical methods used, and treatment outcomes. The patients were divided into two groups, according to death (Group 1) or survival (Group 2), and the two groups were compared in terms of the specified parameters.

**Results:**

Of the patients, 15 were male (50%) and 15 female (50%); their mean age was 71.4 (29–94) years. Abdominal pain was the chief complaint in all patients (100%) and mean time from pain onset to hospital admission was 21 (1–72) h. In abdominal exploration, total small bowel (SB) ischemia and necrosis was found in 6 (20%) patients and other patients had subtotal SB, segmental SB, segmental SB with colon, or isolated colon ischemia. Treatment in 15 patients (50%) ended in mortality. Mean age (p = 0.038), urea (p = 0.002), AST (p < 0.001), ALT (p < 0.001), mean platelet volume (MPV; p = 0.002), and amylase (p = 0.022) levels in Group 1 were significantly higher versus Group 2, whereas Ca (p = 0.024) and albumin (p = 0.002) levels were significantly lower.

**Conclusions:**

In this study, unlike other parameters that have been shown to be of prognostic significance in mesenteric ischemia, MPV values at presentation were higher among non-survivors than survivors.

## Introduction

Acute mesenteric ischemia (AMI) can result from vascular occlusive or non-occlusive conditions. Non-occlusive mesenteric ischemia is caused by conditions such as hypovolemia, sepsis, and cardiogenic shock, whereas the underlying cause of ischemia in 70–80% of cases with AMI is the occlusion of the superior mesenteric artery, caused by embolism or thrombosis [1]. Irreversible changes in the bowel mucosa occur within 6 h in the case of acute arterial occlusion, leading to the disruption of the mucosal barrier, which subsequently allows bacterial translocation, peritonitis, sepsis, and rapid progression to multiple organ failure. Bowel ischemia and necrosis develop rapidly due to a lack of sufficient time to develop collateral circulation, particularly in cases with embolism.

Despite advances in diagnosis, treatment, and post-operative care in recent years, AMI still has a high mortality rate, ranging between 40 and 70% [2]. The most important causes of the high mortality include delayed presentation, non-specific clinical findings, lack of simple biochemical parameters that could be routinely used to diagnose the condition early, and time loss while performing tests for differential diagnosis in patients who are not immediately suspected to have AMI at presentation [3]. From a different perspective, if it is impossible to diagnose AMI in the early period in most patients, it becomes more important that parameters be determined that would be useful to predict the disease course at the time of diagnosis.

The aim of the present study was to evaluate the outcomes of surgical treatment over a period of 5 years in patients who underwent surgery with a diagnosis of AMI and to investigate the parameters of prognostic significance.

## Materials and methods

The hospital records of 30 patients who underwent surgical intervention due to acute mesenteric ischemia in the Department of General Surgery, Sakarya University Faculty of Medicine between January 2008 and December 2012 were reviewed retrospectively. The records were investigated regarding demographic data, presence of co-morbid diseases, presenting complaints, time elapsed between symptom onset and hospital admission, laboratory findings at admission, findings at surgical exploration, surgical methods used, and treatment outcomes. The patients were divided into two groups, according to death (Group 1) or survival (Group 2), and they were compared in terms of the specified parameters.

Among the parameters in complete blood counts, leukocytes (WBC), hematocrit (Htc), hemoglobin (Hb), mean platelet volume (MPV), and total platelet count (PC) were evaluated. Among the biochemical parameters, urea, creatinine, sodium (Na), potassium (K), calcium (Ca), chlorine (Cl), aspartate amino transferase (AST), alanine amino transferase (ALT), gamma glutamyl transferase (GGT), alkaline phosphatase (ALP), total bilirubin, albumin, and amylase were evaluated.

Survivors in whom more than 2 years had elapsed since their operation were contacted by phone to obtain their latest condition.

In total, 21 variables were compared between the groups. Student’s *t*-test and Fischer’s exact test were used for comparison between subgroups. Statistical analyses were performed using the SPSS software (ver. 16.0; SPSS, Inc., Chicago, IL, USA). P values < 0.05 were considered to indicate statistical significance.

## Results

Of the patients, 15 were male (50%) and 15 female (50%); their mean age was 71.4 (29–94) years. Of the patients, 22 (73.3%) had a history of comorbid disease and cardiovascular disorders were the most common (*n* = 16; 72.7%). Abdominal pain was the chief complaint in all patients (100%) and mean time from pain onset to hospital admission was 21 (1–72) h. All patients underwent computed tomography (CT) of the abdomen and the use of intravenous contrast agent was avoided in 5 (16.6%) patients due to impaired renal function (creatinine > 2.5).

Hemogram and biochemical analysis results of all patients are presented in Table 1.

**Table 1:** Hemogram and biochemical analysis results of all patients

**Parameters**	**All patients (n = 30)**	**Death (n = 15)**	**Survival (n = 15)**	**p**
Hematocrit (%)	40.3	38.7	41.9	>0.05
Hemoglobin (g/dl)	13.4	12.8	14.0	>0.05
Leukocyte (/μL)	16.043	18.046	14.040	>0.05
Platelet (/μL)	256.563	240.193	272.933	>0.05
MPV	8.4	9.01	7.80	0.002
Urea	76.3	102.9	51.4	0.002
Creatinine (mg/dL)	1.52	1.67	1.06	>0.05
Na	136.6	136.3	137.3	>0.05
K	4.2	4.4	3.9	>0.05
Ca	8.1	7.6	8.6	0.024
Cl	102.5	101.0	103.8	>0.05
AST (U/L)	55.63	89.9	21.3	<0.001
ALT (U/L)	60.5	100.1	20.8	<0.001
GGT	35.5	36.7	34.7	>0.05
ALP	84.6	81.0	88.9	>0.05
T.Bilirubin	1.3	1.6	0.9	>0.05
Albumin	3.2	2.6	3.8	0.002
Amylase	137.6	214.0	73.0	0.022

In the abdominal exploration, 6 (20%) patients were found to have diffuse small bowel (SB) ischemia and necrosis, and 17 (56.6%) had subtotal (> 100 cm) SB ischemia; of the 17, 8 (47.0%) had right colonic ischemia. Five (16.6%) patients only had segmental SB ischemia and necrosis (<100 cm) and 1 (3.3%) patient had isolated right-sided colonic ischemia and necrosis. The operation was terminated without performing further intervention in patients suffering from diffuse SB ischemia and necrosis (total necrosis), whereas various resections were performed in the remaining 23 patients (76.6%): 9 (9/23; 39.1%) patients underwent subtotal SB resection, 8 (8/23; 34.7%) underwent subtotal SB resection plus right hemicolectomy, 5 (5/23; 21.7%) underwent segmental SB resection, and 1 (1/23; 4.3%) patients underwent a right hemicolectomy.

One patient (3.3%) was admitted to the hospital 1 h after the onset of abdominal pain and CT scans showed occlusion of the superior mesenteric artery (SMA). This patient subsequently underwent an embolectomy due to the presence of subtotal ischemic changes (dark color in the affected organs, decreased peristalsis, no pulses in the small mesenteric arteries) in the SB but without necrosis.

Demographic features and exploration findings of the patients are presented in Table 2.

**Table 2:** Demographic features and exploration findings

**Parameters**	**All patients (n = 30)**	**Death (n = 15)**	**Survival (n = 15)**	**p**
Age		78.07	64.80	0.038
Co-morbid disease	22	12	10	>0.05
Diffuse SB ischemia	5	5	—	
Diffuse SB + colon ischemia	1	1	—	
Subtotal SB ischemia	10	4	6	
Subtotal SB + colon ischemia	8	4	4	
Segmental SB ischemia	5	1	4	
Segmental SB + colon ischemia	—	—	—	
Isolated colon ischemia	1	—	1	
Colon ischemia (+)	10	5	5	>0.05

The treatment resulted in mortality in 15 patients (50%) (6 of them had total necrosis and underwent only exploratory laparotomy) and there were 15 survivors (50%), discharged after a mean follow-up of 5 days [3-12]. In a mean follow-up period of 21 months (3–49), 2 (13.3%) patients died for reasons other than recurrence of mesenteric ischemia. Among the remaining 13 patients, only 1 (1/13; 7.6%) patient, who initially underwent an embolectomy, was re-admitted due to the recurrence of mesenteric ischemia at 13 months, and the patient subsequently underwent a subtotal SB resection.

In comparisons of the non-survivors (group 1, *n* = 15) and survivors (group 2, *n* = 15), mean age (p = 0.038), urea (p = 0.002), AST (p = 0.001), MPV (p = 0.002), and amylase (p = 0.022) levels in Group 1 were significantly higher than in Group 2, whereas Ca (p = 0.024) and albumin (p = 0.002) levels were significantly lower. No significant difference was found between the groups in terms of other parameters.

## Discussion

Acute mesenteric ischemia is among those rare clinical conditions for which no significant improvement has been achieved in the prognosis, despite advances in diagnosis and treatment. Severe abdominal pain with a sudden onset and indistinct physical examination findings are common traits that could be considered to be classic for AMI. However, this is not straightforward and requires experience to consider the diagnosis of AMI based on this clinical picture. Time, which is the strongest and the most valuable factor affecting prognosis, has already been lost in late-presenting patients [4]. The need for radiological imaging of a mesenteric vascular tree for a definitive diagnosis (using multi-slice CT, multi-detector row CT angiography, or conventional angiography), and the fact that these methods are not always readily available consume valuable time in patients presenting at an early stage [1]. In the current study, only one patient (time to admission = 1 h) did not show transmural ischemia and treatment other than surgical resection was possible.

Various biochemical parameters have been investigated for diagnosing acute mesenteric ischemia earlier. Leukocytosis, metabolic acidosis, elevated serum amylase levels, high lactate (L and D stereoisomers), and high D-dimer levels can be found in the presence of AMI. Studies have shown that these findings are not useful in the early diagnosis of AMI and can even be elevated in acute abdominal conditions other than AMI due to their low sensitivity [5-9]. Based on the assumption that mucosa-derived enzymes could be used in the early diagnosis of AMI, considering that ischemia begins from the mucosa, several enzymes, such as intestinal fatty acid binding protein and alpha-glutathione S transferase, have been tested in some studies, which reported limited utility [10]. Leukocytosis, metabolic acidosis, and elevated amylase levels were common findings in the current study; however, these were considered to be expected results considering the long mean time to presentation. D-dimer and mucosa-derived enzymes are not routinely studied in patients presenting to our clinic with abdominal pain.

Predictive factors affecting mortality in patients with AMI upon admission to the hospital have been analyzed in various studies, which yielded different results for many parameters. Aliosmanoglu et al. [11] reported a positive correlation between mortality and leukocytosis, whereas Mamode et al. [12] reported a correlation with leukopenia. Sitges-Serra et al. [13] associated high urea-creatinine levels with poor prognosis, and Aktekin et al. [3] reported that the same parameters were higher in survivors. Acosta-Merida et al. [14] reported an association between hyperamylasemia and massive necrosis, whereas Unalp et al. [15] did not report any association between hyperamylasemia and poor prognosis. Huang et al. [16] reported an association between elevated aspartate aminotransferase (AST) levels and the mortality, and Aktekin et al. [3] reported an association with elevated alanine aminotransferase (ALT) levels. In the current study, urea, AST, ALT, and amylase levels in Group 1 were found to be significantly higher than Group 2, whereas Ca and albumin levels were significantly lower. These results are predictable, considering that late presentation was a common feature of the patients.

Many studies have described advanced age and colonic ischemia accompanying small bowel ischemia as factors indicating poor prognosis [14-17]. In the current study, the mean age in Group 1 was higher than Group 2, consistent with literature reports. However, accompanying colonic ischemia had no effect on prognosis. This could be explained by the small number of patients presenting with colon involvement in the current study compared with in previous reports.

Platelets play a critical role in the regulation of blood flow and thrombogenic cascades. MPV is a marker of the size and activation of platelets, and elevated levels of MPV reflect increased production and activation of platelets. Large platelets possess higher metabolic and enzymatic activity, and show higher thrombogenic potential [18]. Several molecules released from activated platelets, such as P-selectin and thromboxane A2, contribute to thrombus formation; activated platelets also attach to endothelium and up-regulate the expression of adhesions molecules [19]. It was thought that increased MPV could be associated with increased vascular inflammation and thrombogenicity, and a direct association has been shown between increased MPV and acute thrombotic events, such as acute myocardial infarction, unstable angina, and stroke [20-22]. Furthermore, increased MPV was found to be an independent predictor factor of mortality in ischemic vascular events, recurrent myocardial infarction, and coronary artery disease [23].

No published study has examined the relationship between MPV and AMI. AMI is a cardiovascular disease in origin, although its consequences affect predominantly the gastrointestinal system. As a matter of course, a relationship between AMI and increased MPV is considered to indicate increased thrombogenic activity. In the current study, MPV in Group 1 was significantly higher than in Group 2.

However, it would not be appropriate to consider that this result indicates that “increased MPV is a predictive factor for prognosis in AMI,” because a high MPV is found in other atherosclerosis-related conditions (such as diabetes mellitus, hypertension, hypercholesterolemia, smoking, and obesity) [24]. High mean age and the presence of co-morbid conditions related to the cardiovascular system in most of our patients suggest that these patients might have had a high MPV before the development of AMI.

Considering the significantly higher MPV in Group 1 in the current study: 1) MPV could be used to predict the potential for vascular damage in other organs, such as the liver and kidneys (that is, to identify candidate multi-organ failure patients), and 2) because it reflects a tendency for thrombosis, MPV could be useful to justify re-operation when a second-look decision could not be made otherwise.

In conclusion, AMI continues to have a high mortality rate despite advances in diagnosis, treatment, and post-operative care. MPV can be beneficial in predicting patients with poor prognosis and in the planning of re-operations.

## Competing interests

The authors declare that they have no competing interests.

## Authors’ contributions

FA, YA and OVO contributed to study design. YA, OY and YU contributed to data collection. FA and YA contributed to data analysis and writing. All authors read and approved the final manuscript.
